# Comparison of SARS-CoV-2 related in-hospital mortality, ICU admission and mechanical ventilation of 1.4 million patients in Germany and Switzerland, 2019 to 2022

**DOI:** 10.1007/s15010-024-02412-9

**Published:** 2024-10-17

**Authors:** Cathrin Kodde, Sven Hohenstein, Irit Nachtigall, Yvonne Cavalli, Reto Schuepbach, Raphael Graf, Andreas Bollmann, Ralf Kuhlen

**Affiliations:** 1 Department of Infectious Diseases, Respiratory Medicine and Critical Care, Charité–Universitaetsmedizin Berlin, Berlin, Germany; 2 Department of Respiratory Diseases, Lungenklinik Heckeshorn, Helios Hospital Emil-Von-Behring, Berlin, Germany; 3 Helios Health Institute, Berlin, Germany; 4 Department of Infectious Diseases and Infection Prevention, Helios Hospital Emil-Von-Behring, Berlin, Germany; 5 Faculty of Medicine, MSB Medical School Berlin, Berlin, Germany; 6 University Hospital Zurich, Zurich, Switzerland; 7 Initiative Quality Medicine, Berlin, Germany; 8 Institute of Intensive Care Medicine, University Hospital Zurich and University Zurich, Zurich, Switzerland; 9 3M Health Information Systems, Neuss, Germany; 10 Department of Electrophysiology, Heart Centre Leipzig at University of Leipzig, Leipzig, Germany

**Keywords:** SARS-CoV-2, ARDS, Surveillance, Public health, Respiratory infections, Critical care

## Abstract

**Purpose:**

In the 2020 emergence of SARS-CoV-2, global response lacked unified treatment and surveillance, resulting in diverse impacts due to varied healthcare resources and national guidelines. Germany and Switzerland curbed the virus initially by promptly tracking and testing, bolstered by strong governmental capacity. This study aimed to assess country-specific healthcare disparities and their impact on ICU admission rates, mechanical ventilation, and in-hospital mortality.

**Methods:**

To enhance healthcare quality using real-world data, the “Initiative of Quality Medicine” (IQM) was established. Pseudonymised routine data from participating hospitals, during 01/01/2019–31/12/2022, was retrospectively analysed, focusing on patients with SARI ± SARS-CoV-2-infection (U07.1). Cohorts were matched based on various factors and multivariable analyses included logistic regression.

**Results:**

1.421.922 cases of SARI ± U07.1 involving 386 German and 41 Swiss hospitals were included. Patients in Germany were older (mean: 69.4 vs. 66.5 years) and had more comorbidities than in Switzerland (p < .001). Patients in Germany were also more likely to be treated on ICU (28% vs. 20%, OR 1.5 95% CI 1.5–1.6, p < .001) and mechanically ventilated (20% vs. 15%, OR 1.4, 95% CI 1.4–1.5, p < .001). The in-hospital mortality was significantly higher in Germany than in Switzerland (21% vs. 12%, OR 2.0, 95% CI 1.9–2.0, p < .001). Matched cohorts showed reduced differences, but Germany still exhibited higher in-hospital mortality. Discrepancies were evident in both pre-pandemic and pandemic analyses, highlighting existing disparities between both countries.

**Conclusion:**

IQM data from Swiss and German hospitals reveals country-specific differences in SARI ± U07.1 outcomes, highlighting higher in-hospital mortality in Germany, with uncertain causes suggesting varied treatments and resources.

## Introduction

At the onset of the global emergence of the severe acute respiratory syndrome coronavirus 2 (SARS-CoV-2) in 2020, treatment regimens, guidelines and surveillance methods were scarce, prompting the need to establish them from scratch. Even though the World Health Organization (WHO) published treatment suggestions, there was a lack of cooperation among countries in implementing common guidelines. A challenge during this time was that countries, even within Europe, had different healthcare resources and followed their own national guidelines. Consequently, the impact of the pandemic varied significantly across nations. Germany and Switzerland, notably, demonstrated effective measures in curbing the spread of the virus by responding early through case tracking and testing, which was supported by strong governmental capacity to implement health policies and national guidelines [1]. Particularly Germany gained recognition for its low mortality rate among reported cases during the initial phase of the pandemic. As the pandemic progressed, incidence, case fatality rate and clinical outcome began to differ with lower COVID-19 related deaths in Switzerland. Recently, studies emerged stating that Germany’s mechanical ventilation rate and its in-hospitality mortality rate exceeded that of other countries [1–4].

Continuous surveillance of epidemiological trends such as case counts, intensive care unit (ICU) capacities, and in-hospital mortality is crucial in managing pandemics. Additionally, it is essential to monitor severe acute respiratory infections (SARI) during local outbreaks, not only related to SARS-CoV-2, for both proactive and reactive responses enabling effective public health measures, healthcare planning, and strategies to mitigate the impact on individuals and the healthcare system as a whole.

In both countries, Germany and Switzerland, statutory surveillance systems exist. In Germany, the Robert Koch-Institute collects data of acute respiratory infections through different methods, including information from sentinel hospitals, outpatient clinics, self-monitoring and wastewater analysis [5]. In Switzerland, the data collection is more decentralised but relies on similar methods [6, 7].

However, not only are these governmental structures in place. To manage quality of hospital care based on real world administrative data, the “Initiative of Quality Medicine” (Initiative Qualitaetsmedizin; IQM) was founded in 2008 [8]. This association consists of a total number of 500 hospitals from Switzerland and Germany. Members of the IQM in Germany and Switzerland collectively provide care for roughly 7.8 million hospitalized patients each year. This accounts for nearly 40% of inpatients in Germany and approximately 35% of inpatients in Switzerland.

Using aggregated data obtained from a set of routine data provided by IQM, we conducted an in-depth analysis of clinical outcomes of SARS-CoV-2 and severe acute respiratory infection in both countries. The aim of the study was to unveil country-specific characteristics related to ICU admission rates, mechanical ventilation, and in-hospital mortality for SARI ± U07 and what factors might contribute to these differences. The findings are also analysed in the broader context of societal impact.

## Methods

In the framework of the IQM, participating hospitals regularly submit anonymised routine claims data to 3M Health Information Systems, Neuss, Germany for analysis of well-defined IQM quality indicators. Results of the routine data-based quality indicators are calculated and subsequently made openly accessible. For the actual analysis, we used a subset of the aggregated administrative data of patients who had a full inpatient treatment, a diagnosis of SARI (ICD-10 codes J.09–J.22) and/or COVID-19 (U07.1). Patients without lab-confirmed detection of SARS-CoV-2 (U07.2) were not analysed in this study. Data was provided by 41 Swiss hospitals and 386 German hospitals from 01-01-2019 until 31-12-2022 and was retrospectively analysed. We defined two time periods: pre-pandemic from 01/01/2019 until 31/12/2019 and pandemic from 01-01-2020 until 31-12-2022. Statistics for the following treatments and outcomes were compiled: SARS-CoV-2 infection (ICD-10 U07.1), intensive care, mechanical ventilation, in-hospital mortality, length of stay (days in hospital), duration of mechanical ventilation (hours) and type of clinic (university hospital, tertiary and primary care, specialty hospital). For a better comparison, we matched cohorts according to age, sex, hospital type, SARS-CoV-2 infection, Elixhauser Comorbidity Index (ECI) and Hospital Frailty Risk Index. We report statistics for ECI. For the weighted ECI, the AHRQ algorithm was applied [9]. The ECI consists of a list of 30 comorbidities, such as diabetes, hypertension, congestive heart failure, and others. Each comorbidity is assigned a score based on its presence or absence in the claims data set. The scores are used to provide a quantitative measure of the patient’s overall comorbidity burden. The Hospital Frailty Risk Index score is a weighted sum of 109 comorbidities (defined as three-digit ICD-10 codes) for elderly patients. The authors distinguish three risk groups who are at a greater risk of adverse outcomes based on the score: Low risk (score < 5), Intermediate risk (score 5–15), High risk (score > 15) [10].

Statistical analyses were performed in the R environment for statistical computing (version 4.0.2, 64-bit built). For the description of the patient characteristics, we employed chi-square test for categorical variables and two-sample t-test for numeric variables. We report proportions, means, standard deviations, and *p* values. Outcomes of in-hospital care as well as proportions of cohorts were analysed via logistic regression for binary variables and linear regression for numeric variables. We report proportions, means, standard deviations, medians, interquartile ranges, and *p* values. Here, “Difference” refers to odds ratios and linear coefficients, respectively.

For the analyses of the outcome variables length of stay and duration of mechanical ventilation, we log-transformed the dependent variables due to their skewed distributions. Multivariable analyses of outcomes were performed via logistic regression. Categorical variables entered the analyses as simple-coding contrasts while the continuous variable age was centred at its mean. All continuous variables were scaled to unit variance.

Analyses were done for the whole cohort as well as for a subset of a matched cohort (with a ratio between German cases and Swiss cases of 1) for which we employed nearest neighbour matching without replacement on the propensity score estimated via logistic regression with respect to age, sex, SARS-CoV-2 infection, hospital type, ECI, and frailty index.

## Results

Data derived from 386 German hospitals, accounting for 20.46% of all German hospitals (n = 1887). 41 out of 276 (14.86%) Swiss hospitals provided data for the analysis. With a total of 7.8 million cases (2022) the ÌQM data represents roughly 40% of the inpatients in Germany and 35% in Switzerland, respectively. For the whole study period, a total of 1.421.922 cases were considered for the diagnosis of SARI (without U07.1) and SARI with U07.1 (see Table 1).

**Table 1:** Number of cases according to country

Diagnosis	Switzerland n = 118.851	Germany n = 1.303.071	Odds ratio	95% CI	p-value
SARI	89.681 (75%)	1.087.150 (83%)	1.6	1.6–1.7	< 0.001
SARI & U07.1	29.170 (25%)	215.921 (17%)	0.61	0.60–0.62	< 0.001

SARI and U07.1 (without SARI) patients in Germany were significantly older compared to patients in Switzerland (mean age 69.4 years vs. 66.5 years, p < 0.001). In both countries, more females were treated (Switzerland: 17.694 (61%); Germany: 124.056 (57%), p < 0.001). Patients in Germany were in a more compromised health condition as reflected by higher ECI and Hospital Frailty Risk Index. Patients in Germany had a mean ECI of 11.5 (standard deviation (SD) 10.8) compared to 9.2 (SD 11.5) in Switzerland (p < 0.001). The Hospital Frailty Risk Score that is widely used in elderly patients yield similar results (mean score of 8.3 vs. 5.2; SD 6.9 vs. 5.2; p < 0.001). Table 2 shows the baseline characteristics of the patient population for each country.

**Table 2:** Baseline characteristics for SARI and U07.1, mean (SD); n (%), Welch Two Sample t-test; Pearson’s Chi-squared test

Baseline characteristics	Switzerland n = 29.170	Germany n = 215.921	Difference (95% CI)	p-value
Age	66.5 (17.6)	69.4 (17.6)	− 2.9 (− 3.1, − 2.7)	< 0.001
Age group				< 0.001
≤ 17 years	384 (1.3%)	2.625 (1.2%)		
18 − 59 years	8.766 (30%)	52.065 (24%)		
60 − 69 years	5.760 (20%)	36.749 (17%)		
70 − 79 years	6.727 (23%)	47.168 (22%)		
≥ 80 years	7.533 (26%)	77.314 (36%)		
Sex				< 0.001
Male	11.476 (39%)	91.860 (43%)		
Female	17.694 (61%)	124.056 (57%)		
Not available	0	5		
Hospital type				< 0.001
Specialty hospital	72 (0.2%)	2.707 (1.3%)		
Primary care	14.544 (50%)	145.286 (67%)		
Tertiary hospital	2.194 (7.5%)	42.492 (20%)		
University hospital	12.360 (42%)	25.436 (12%)		
Elixhauser comorbidity index				< 0.001
< 0	4.162 (14%)	23.555 (11%)		
0	5.845 (20%)	29.039 (13%)		
1–4	2.087 (7.2%)	11.060 (5.1%)		
≥ 5	17.076 (59%)	152.267 (71%)		
Elixhauser comorbidity score	9.2 (11.0)	11.5 (10.8)	− 2.3 (− 2.4, − 2.2)	< 0.001
Hospital frailty risk index				< 0.001
< 5	17.768 (61%)	87.550 (41%)		
5–15	9.674 (33%)	92.756 (43%)		
> 15	1.728 (5.9%)	35.615 (16%)		
Hospital frailty risk score	5.2 (5.2)	8.3 (6.9)	− 3.0 (− 3.1, − 3.0)	< 0.001

SARI Patients with concomitant U07.1 were more likely to be treated on an ICU in Germany (28% vs. 20%, OR 1.5 95% CI 1.5–1.6, p < 0.001) and more likely to be mechanically ventilated (20% vs. 15%, OR 1.4, 95% CI 1.4–1.5, p < 0.001). However, patients in Switzerland were mechanically ventilated for longer periods than in Germany (290.5 h, median 192 h [inter-quartile range: 64–408 h] vs. 268.2 h, median 153 [43–362 h], 95% CI − 33– − 12, p < 0.001).

The in-hospital mortality was significantly higher in Germany than in Switzerland (21% vs. 12%, OR 2.0, 95% CI 1.9–2.0, p < 0.001). To identify risk factors for the in-hospital mortality a multivariable analysis was performed and showed the known risk factors age, mechanical ventilation, intensive care treatment, and higher comorbidities. However, when analysed by country, treatment in Germany was an independent risk factor for higher mortality as well (OR 1.53, 95% CI 1.47–1.60, p < 0.001 see Table 3).

**Table 3:** Results of multivariable analyses of In-hospital mortality

Variable	OR (95% CI)	p-value
Male sex	1.25 (1.22–1.28)	< 0.001
Age	6.79 (6.59–6.99)	< 0.001
Specialty hospital vs. primary care	0.95 (0.86–1.05)	0.327
Tertiary hospital vs. primary care	1.20 (1.16–1.23)	< 0.001
University hospital vs. primary care	1.20 (1.16–1.24)	< 0.001
Germany vs. switzerland	1.53 (1.47–1.60)	< 0.001
Intensive care	2.08 (2.00–2.17)	< 0.001
Mechanical ventilation	3.60 (3.44–3.76)	< 0.001
Elixhauser comorbidity score	1.60 (1.56–1.63)	< 0.001
Frailty risk score	1.10 (1.08–1.12)	< 0.001

As stated earlier, differences in the baseline characteristics for each country’s patient population exist, especially for age distribution, comorbidities, and frailty. In an attempt to adjust for these confounding variables, we did a statistical analysis with a subset of the total cohort by synthetizing a matched cohort (all 118.851 Swiss cases and 118.851 matched German cases; SARI ± U07.1), which resulted into well-balanced covariates depicted in Table 4.

**Table 4:** Baseline characteristics of matched cohorts. Mean (SD); n (%), Welch Two Sample t-test; Pearson’s Chi-squared test

Baseline characteristics	Switzerland N = 90,320	Germany N = 90,320	Difference (95% CI)	p-value
Age	59.5 (28.0)	58.7 (28.0)	0.71 (0.45–0.97)	< 0.001
Age group				< 0.001
≤ 17 years	13,065 (14%)	13,469 (15%)		
18 − 59 years	19,646 (22%)	20,115 (22%)		
60 − 69 years	14,737 (16%)	15,284 (17%)		
70 − 79 years	19,610 (22%)	18,150 (20%)		
≥ 80 years	23,262 (26%)	23,302 (26%)		
Sex				0.072
Male	37,014 (41%)	36,635 (41%)		
Female	53,306 (59%)	53,681 (59%)		
N/A	0	4		
SARS-CoV-2 infection	29,170 (32%)	28,955 (32%)		0.3
Hospital type				< 0.001
Specialty hospital	144 (0.2%)	127 (0.1%)		
Primary care	41,311 (46%)	42,606 (47%)		
Tertiary care	6919 (7.7%)	6611 (7.3%)		
University hospital	41,946 (46%)	40,976 (45%)		
Elixhauser comorbidity index				< 0.001
< 0	8868 (9.8%)	8634 (9.6%)		
0	21,204 (23%)	21,722 (24%)		
1–4	6491 (7.2%)	6001 (6.6%)		
≥ 5	53,757 (60%)	53,963 (60%)		
Elixhauser comorbidity score	10.6 (12.3)	10.1 (11.3)	0.53 (0.42–0.63)	< 0.001
Hospital frailty risk index				< 0.001
< 5	52,879 (59%)	53,631 (59%)		
5–15	30,971 (34%)	31,111 (34%)		
> 15	6470 (7.2%)	5578 (6.2%)		
Hospital frailty risk score	5.6 (5.5)	5.4 (5.2)	0.19 (0.14–0.24)	< 0.001

Clinical outcomes such as intensive care utilization, mechanical ventilation, or the duration of ventilation were very similar to the findings in the unmatched cohort. Both, the ECI and Hospital Frailty Risk Score were slightly lower in Germany than Switzerland after matching cohorts (ECI of 10.1 vs. 10.6, Hospital Frailty Risk Score of 5.4 vs. 5.6). Nonetheless, the difference in in-hospital mortality decreased but was still significant higher in Germany (12% vs. 9.0%, OR 1.41, 95% CI 1.3–1.4, p < 0.001). Treatment in Germany was also an independent risk factor for in-hospital mortality in a multivariable analysis (now also including SARS-CoV-2 infection as a predictor; OR 1.41, 95% CI 1.37–1.46, p < 0.001).

Throughout the pandemic, the regulations for restriction and different measures to prevent further spreading of the infection differed between Germany and Switzerland, which might have influenced the clinical characteristics. We therefore analysed the data before 2020, which only included the diagnosis of SARI and formed matched cohorts for this cohort as well. In the unmatched cohorts, German patients were significantly older and had more comorbidities than the Swiss study population (64.3 years vs. 59.6 years, p < 0.001; ECI 13.7 vs. 10.8, p < 0.001, Hospital Frailty Risk Score 8.3 vs. 5.6, p < 0.001). Pre-pandemic matched cohorts showed comparable results as pandemic cohorts with a marginal decrease in comorbidities for patients treated in Germany below Switzerland’s comorbidities scores (ECI 10.5 vs. 10.8, Hospital Frailty Risk Score 5.4 vs. 5.6,).

SARI patients in the matched pairs multivariable analysis before the pandemic were again more likely to be treated on an ICU in Germany (21% vs. 16%, OR 1.44 95% CI 1.37–1.50, p < 0.001) as well as they were more likely to be mechanically ventilated (14% vs. 10%, OR 1.52 (1.44–1.60), p < 0.001) which is also observed during the pandemic. However, univariate analysis showed that in Switzerland pre-pandemic SARI patients were mechanically ventilated for shorter periods than SARI with U07.1 patients during the pandemic (mean 161.5 h vs. 226.2 h, p = 0.023). In Germany, patients were ventilated for a shorter duration during the pandemic compared to before (matched cohorts; mean 217.5 h vs. 226.2 h, p < 0.001).

**Fig. 1:**
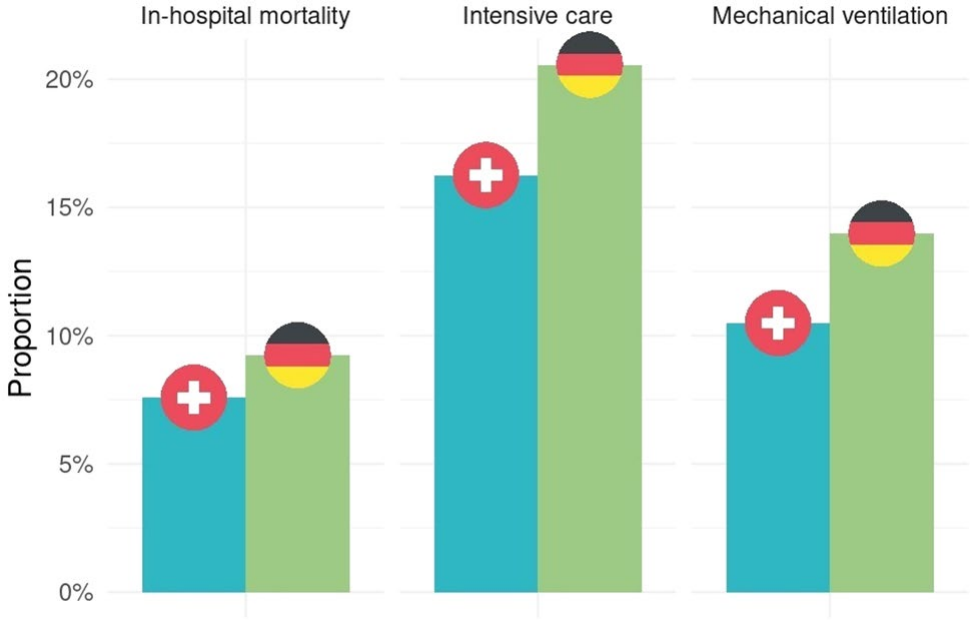
Comparison between Switzerland and Germany for binary outcomes for SARI during Pre-Pandemics. Matched data

**Fig. 2:**
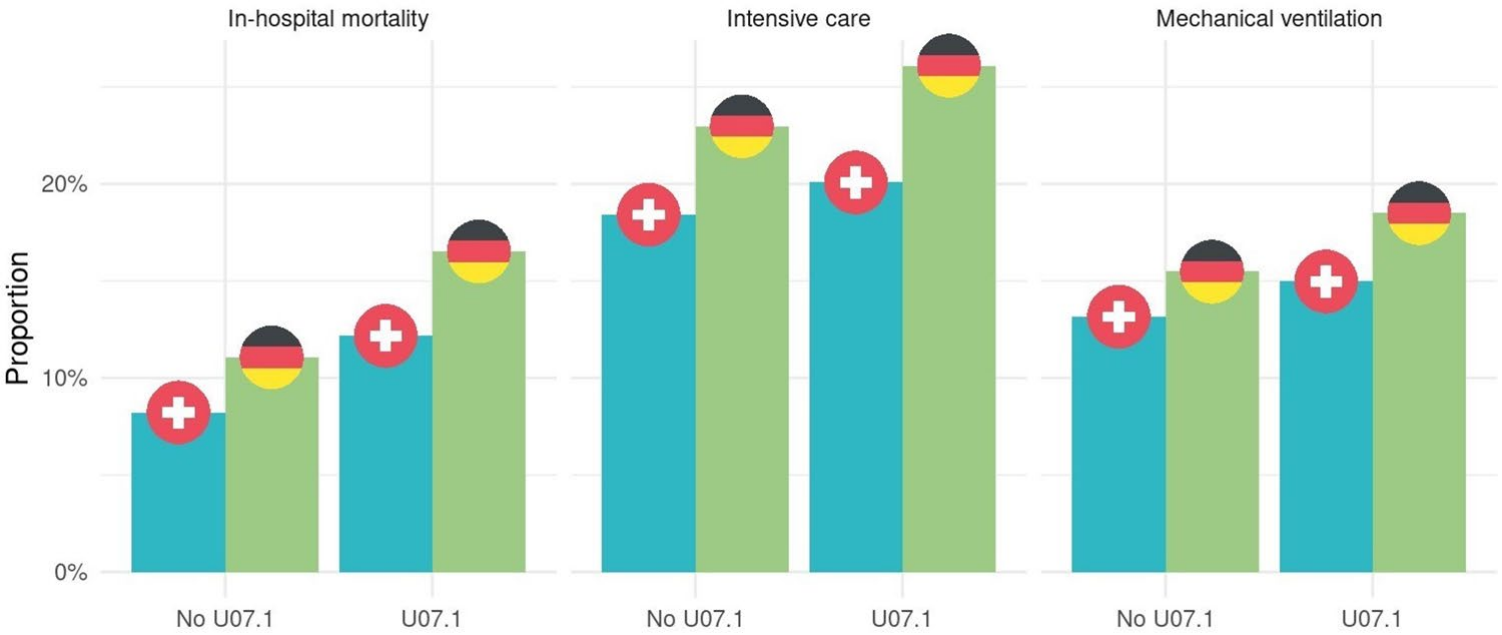
Comparison between Switzerland and Germany for binary outcomes for SARI (with and without U07.1) during COVID-19 pandemic. Matched data

After running a multivariable analysis no statistical significance between both countries for pre-pandemic mechanical ventilation was observed (regression coefficient 0.06 95% CI − 0.01–0.13, p = 0.087). The in-hospital mortality of SARI differed between Germany and Switzerland also before the onset of pandemic with Germany showing a reliably higher mortality than Switzerland for SARI (9.2% vs. 7.6%, OR 1.18, 95% CI 1.11–1.26, p < 0.001). Figure 1 and Fig. 2 show the difference of the binary outcomes pre-pandemic and during pandemic for SARI (with and without U07.1). When comparing pre-pandemic outcomes to the pandemic period, we were able to demonstrate that a disparity between the two countries existed before and was persistent during the pandemic.

## Discussion

The in-hospital mortality rate of COVID-19 emerged as a surrogate marker during the pandemic both nationally and internationally to assess the status of any country’s healthcare situation. However, conducting thorough analyses of clinical outcomes and in-hospital mortality remained infrequent and challenging due to numerous and very heterogeneous influencing factors. In our study, we were able to analyse claims data of a wide range from hospitals in Switzerland and Germany. The main finding of this analysis was that in Germany ICU utilization and mechanical ventilation were more frequent compared to Switzerland, whereas in-hospital mortality rate was lower in Switzerland with similar demographic and health system characteristics.

Our findings are consistent with a recently published study by Karagiannidis et al., which analyses the clinical course of mechanically ventilated patients exclusively in Germany [4]. Karagiannidis et al. reported a high in-hospital mortality rate and mechanical ventilation rate and provided possible explanations for these findings, e.g. high rate of older patients and large capacity of ICU beds in Germany. A key strength of our study is the direct comparison and statistical analysis of SARI cases with and without SARS-CoV-2 in two different yet comparable countries.

During the first wave, Germany’s response to the pandemic was generally praised for its robust healthcare system, widespread testing, and efficient contact tracing with a comparatively low count of COVID-19-related deaths [1, 3]. Possible explanations for this observation included the well-prepared German healthcare system with a high number of ventilators, ICU beds and medical doctors in international comparison [11–13].

In anticipation of a possible medical shortage and necessity for triage of patients, each country published guidelines on how to treat COVID-19 patients under medical resource scarcity [14, 15]. Both guidelines were similar but a few differences were noted. In Germany, the central statement for maximizing benefit was that as many patients as possible should be treated for COVID-19 and deaths should be minimized. Switzerland had a similar statement saying that its central benefit should be preserving as many lives as possible [15–17]. Regarding ICU admissions, the Swiss guideline had stricter admissions criteria in case of a possible triage regarding patients’ comorbidities and age-related clinical frailty score than Germany [15, 17, 18]. Additionally, both countries’ guidelines emphasise the importance of discussing the patients will in advance in case of COVID-19 infection and if complications occur (e.g. resuscitation, shift to ICU or best supportive care). Even though, it is stated in both guidelines, advance care planning (APC) has been widely promoted and firmly established only in Switzerland over the last decade [19]. One of the key strengths of ACP is to enhance patient-centered care by ensuring that medical decisions align with the individual’s values and preferences [20, 21]. Additionally, ACP in Switzerland has been associated with a reduction in unnecessary medical interventions, such as hospitalizations and intensive treatments at the end of life, allowing patients to receive care in their preferred setting. These outcomes not only improve quality of life for patients but also contribute to more efficient use of healthcare resources. Switzerland’s relatively liberal end-of-live choices may have resulted in less hospital and ICU admissions related to COVID-19 because of intensified and proactive APC for elderly or critical ill patients.

Besides guidelines in case of resource scarcity, treatment recommendations for the so far unknown SARS-CoV-2 were published. They were subject to a steady change and were frequently adapted and changed profoundly during the pandemic. This resulted in a lack of consistent recommendations and universal guidelines.

For example, unlike Germany, Switzerland followed a more conservative way of respiratory support after the first wave with the early application of High Flow nasal cannula (HFNC) or non-invasive ventilation (NIV) on intermediate care rather than an early transfer for intubation on ICU [22]. In Germany, treatment regimen in the beginning of the pandemic recommended an early intubation even though the evidence was weak [2, 23]. Growing evidence suggests that mechanical ventilation might have an adverse impact on SARS-CoV-2-induced Acute Respiratory Distress Syndrome (ARDS) [24, 25]. This finding is in accordance with our study, where Swiss patients were significantly less mechanically ventilated and treated on ICU with a lower in-hospital mortality rate than patients in Germany.

Notably, the in-hospital mortality rate among the matched cohorts in Germany persisted at a higher level, suggesting factors beyond age and comorbidities. While there was a significantly lower ECI and Frailty Index of patients in Germany, the difference was numerically small and can be attributed to the large study population. Given that a majority of deaths occur in hospitals, disparities are primarily attributed to varying medical care practices, where fast intubation and mechanical ventilation potentially lead to poorer outcomes [2, 26].

During the pandemic, Germany merely experienced times where ICU capacity was reached [26], where at some point due to the high number of infection in their surrounding borders like France and Italy some Swiss cantons faced briefly an excess utilization of ICU beds [1]. This could potentially explain why there might have been restrictions to overuse ICU beds and mechanical ventilation in Switzerland. Contrarily, Germany did not require any restrictions due to its reputation for having one of the highest ICU beds per population worldwide, providing a somewhat "unlimited" resource capacity. Additionally, in Germany, physicians follow the concept of “clinical freedom” granting considerable autonomy in their decision-making in patient care e.g. indication for mechanical ventilation. Swiss doctors do also possess autonomy but might function within a more structured or regulated healthcare environment where recommendations can be implemented and directed on a national scale [6, 18]. Similarly, a research by Karagiannidis et al. critically analysed the high in-hospital mortality and utilization of extracorporeal membrane oxygenation (ECMO) during COVID-19 pandemic in Germany to other countries [27]. In our study, we were also able to show that even before the pandemic, patients with SARI had a higher mortality in Germany than in Switzerland. This finding supports the assumption that in Germany due to the relatively large and easy availability of ICU beds and ventilators without restriction, physicians tend to use them early on [4]. The fact that a higher pre-pandemic in-hospital mortality rate was observed in Germany with a comparable mechanical-ventilation rate suggests that there might be reasons other than the pandemic (e.g., SARI in-patient population, admission policies, and treatment pathways) which should be addressed in further studies.

Prior to the SARS-CoV-2 pandemic, research indicated a link between the level of staffing in critical care settings and patient mortality rates. Specifically, a higher ratio of patients to critical care staff was correlated with poorer patient outcomes, including higher rates of infection transmission, postoperative complications such as respiratory failure and the need for reintubation, and increased mortality. However, there is limited research assessing how critical care staffing affects ICU mortality during a pandemic [28–30]. Staffing could not be directly linked to outcome of COVID-19 patients requiring intensive care [28]. Nevertheless, in some older papers, greater staffing available in Swiss ICU has been reported, potentially contributing to more effective ICU services [31]. A Swiss paper examined the patient-critical care staffing ratio during the first months of the pandemic and reported a daily patient-to-nurse ratio (NPR) ranging from 1.0 to 2.4 with a daily patient-to-physician ratio (PPR) ranging from 4.0 to 6.8. In this study, no correlation between limited critical care staffing resources in Swiss ICUs and either the overall length of stay in the ICU or mortality rates was observed [28]. In Germany, legislation mandates a minimum nurse-to-patient ratio in ICUs, specifying at least 1 nurse per 2.5 patients during the day and 1 nurse per 3.5 patients at night. The actual NPR and PPR in German ICUs during the pandemic are difficult to obtain. Nevertheless, papers suggest that the legal requirements were not always met [32, 33]. The previously mentioned mandate was even temporarily suspended during the beginning of the pandemic to ensure the hospitals workflow but was reinforced from summer 2020 [34]. However, despite this requirement, the persistent shortage of ICU specialists in Germany creates a gap between these regulatory standards and actual staffing levels, potentially compromising the quality of care.

Furthermore, due to the expected scarcity of medical supplies, governmental regulations in both states mandated the postponement of elective procedures to allocate resources for the treatment of COVID-19 patients. In contrast to Germany, Switzerland relaxed its restriction on non-urgent healthcare services already after the first wave [35]. In Switzerland it was observed that elective procedures decreased by approximately 53% during the first wave compared to pre-pandemic years. However, the inpatient treatments recovered almost completely during the following waves [35]. In contrast to that, Germany reinforced the order to postpone elective procedures and hospital admissions without rapid reconstitution of inpatient numbers [36–38].

To assess the ICU occupancy, only COVID-19 cases were recorded, while other illnesses were not taken into account. This resulted in a mismatch of hospital occupancy, with reduced activity of smaller hospitals that conducted many elective procedures before, while specialized tertiary care centres were more heavily burdened due to influx of COVID-19 patients. The total number of cases, especially with regard to ICU occupancy and mechanical ventilation, did not exceed the pre-pandemic numbers overall [38].

At the onset of the pandemic, the primary indicator for the prevailing COVID-19 situation was based solely on the total number of detected SARS-CoV-2 cases. As the pandemic advanced, more experiences were acquired in assessing accurately the hospital occupancy. That led to an expansion of indicators by including COVID-19 related ICU occupancy, case numbers and reproduction number, which measures the average number of secondary infections produced by one infected individual in a susceptible population. The so-called “Corona Ampel/COVID-19 traffic light” was established in Germany and in many countries of the European Union but not in Switzerland.

A limitation of our study is that the data is purely descriptive from a large dataset but detailed analysis of the hospital admission criteria was not collected. That is why the influence of disease severity, SARS-CoV-2 variants, use of steroids, antivirals, or other disease modifying medications is not reported. Additionally, there might be a bias towards insufficient coding which cannot be ruled out completely. However, coded data is primarily taken for reimbursement purposes and can be crosschecked by the payor institutions, we assume that undercoding is not a systemic issue.

To ensure the best quality of care and enhance pandemic preparedness, we suggest that not only case numbers should be considered. The surveillance and inclusion of parameters such as case parameters (age, gender, ECI/Frailty Index), outcomes (in-hospital mortality, intensive care, mechanical ventilation) and durations (length of stay, length of mechanical ventilation) are an asset in monitoring the next pandemic.

## Conclusion

Analysing standardized real-world data of Swiss and German hospitals from the IQM network provide a profound basis for monitoring and comparing clinical outcomes for SARI and COVID-19 (SARI with U07.1). We were able to show country-specific differences in the clinical course of COVID-19, a higher mechanical ventilation rate and in-hospital mortality in Germany. These findings were evident even before the pandemic. The causes for this discrepancy remain uncertain. However, it can be assumed that varying treatment surveillance methods and guidelines, possible healthcare resources and restrictive use of mechanical ventilation play significant roles in the diverse outcome for SARI and U07.1. Further research into the causes of country-specific discrepancies, and potential harmonization of treatment guidelines and healthcare resources could help improve patient outcomes for SARI and COVID-19 in the future.

## Data Availability

The data that support the findings of this study are not openly available due to reasons of sensitivity and data protection rules.
